# The value of multimodal ultrasonography in differential diagnosis of tuberculous and non-tuberculous superficial lymphadenitis

**DOI:** 10.1186/s12893-021-01418-6

**Published:** 2021-12-14

**Authors:** Jie Chu, Ying Zhang, Wenzhi Zhang, Dan Zhao, Jianping Xu, Tianzhuo Yu, Gaoyi Yang

**Affiliations:** Department of Ultrasound, Zhejiang Hospital of Integrated Traditional Chinese and Western Medicine, 208 Huancheng East Road, Downtown District, Hangzhou, 310003 Zhejiang China

**Keywords:** Multimodal ultrasonography, Superficial tuberculous lymphadenitis, Tuberculosis

## Abstract

**Background:**

To investigate the value of multimodal ultrasonography in differentiating tuberculosis from other lymphadenopathy.

**Methods:**

Sixty consecutive patients with superficial lymphadenopathy treated at our hospital from January 2017 to December 2018 were categorized into four types based on the color Doppler ultrasound, five types based on contrast-enhanced ultrasound, and five types based on elastography. Sensitivity and specificity were calculated of all the three imaging, including color Doppler examination, contrast-enhanced ultrasound and one individual multimodal method, for detecting lymph nodes.

**Results:**

A total of 60 patients were included in the final analysis. Of those, Mycobacterium tuberculosis was positive in 38 patients and negative in 22 patients. Among the 38 patients who were positive for Mycobacterium tuberculosis, of which 23 had a history of pulmonary tuberculosis, accounting for 60.53% of the positive cases, and the remaining patients did not combine lesions of other organs. Among the 60 superficial lymph nodes, 63.3% presented with tuberculous lymphadenitis. The sensitivity, specificity, and accuracy of the color Doppler examination were 73.68%, 68.18%, and 71.67%, respectively. The sensitivity, specificity and accuracy of contrast-enhanced ultrasound were 89.47%, 63.64% and 80.00%, respectively. The sensitivity, specificity and accuracy of the elastography were 63.16%, 63.64% and 63.33%, respectively. The sensitivity, specificity and accuracy of one individual multimodal method were 42.11%, 95.45% and 61.67%, respectively. The sensitivity, specificity and accuracy of all modes combined were 100.00%, 27.27% and 73.33%, respectively.

**Conclusion:**

Multimodal ultrasonography has high predictive value for the differential diagnosis of superficial tuberculous lymphadenitis.

## Background

Tuberculosis is a chronic infectious disease caused by the Mycobacterium tuberculosis complex group. Various organs and systems of the human body can develop this disease. Tuberculous lymphadenitis is a common manifestation of extrapulmonary tuberculosis (TB) [1], caused by *Mycobacterium tuberculosis* invading the lymph nodes to cause superficial TB lymphadenitis, with cervical vertebral TB lymphadenitis being the most common [2]. Clinical manifestations include lymphadenopathy, pain tenderness, and sinus formation.

The causes of tuberculous lymphadenitis are highly complex, and differentiating TB lymphadenitis from non-tuberculous mycobacterial infections can be difficult. However, Early diagnosis of TB lymphadenitis is very important. Patients with TB lymphadenitis often have a poor prognosis due to delays in the diagnosis and treatment of TB Lymphadenitis [3]. In addition to palpation and biopsy of lymph nodes, imaging technology has always been an important method for the diagnosis of superficial lymphadenitis, especially ultrasound, as a noninvasive and radiation-free imaging method, has been widely used in clinics [4]. Nowadays, many modes of ultrasonography can be used, including conventional two-dimensional ultrasound, color Doppler ultrasound, ultrasound elastography and contrast-enhanced ultrasound.

China has one of the world’s largest prevalence rates of TB, and learning from an institution where complex evaluative protocols for possible TB lymphadenitis in a stream-lined fashion may benefit management in both China and other countries including India, Indonesia, Pakistan, and the Russia where prevalence continues to be high [5, 6].

This study was conducted to evaluate the value of different ultrasonography methods for observing superficial TB lymphadenitis and diagnosing superficial mycobacteria using multi-modal ultrasonography. We hypothesized that multimodal ultrasonography may have highly predictive in the diagnosis of TB and non-TB superficial lymphadenitis for multimodal molecular imaging combines two or more inspection technologies to overcome the shortcomings of a single imaging mode [7].

## Materials and methods

### Patients

Complete clinical and laboratory data of consecutive superficial lymphadenopathy patients treated at our hospital from January 2017 to December 2018 were collected. Inclusion criteria: (1) there was no gender preference in this study; (2) patient age above 18 years; (3) patients diagnosed with Color Doppler ultrasound examination, Elastography examination, and Contrast-enhanced ultrasound examination successively; (4) patients underwent puncture biopsy after the ultrasound examination. Exclusion criteria: (1) patients with a history of mental illness; (2) patients who had taken anticoagulant drugs in the past week; (3) patients with a history of allergies to eggs, milk, fish or shrimp; (4) patients who had used hormone therapy within the past week; (5) patients with uncompleted medical records. (6) HIV-infected patients. Informed consents were obtained from all preoperative patients. The study protocol was approved by the Institutional Ethics Committee of Zhejiang Hospital of Integrated Traditional Chinese and Western Medicine with the Helsinki Declaration.

### Diagnostic methods

All patients selected in this study underwent a puncture biopsy after ultrasound examination, and the tissues were used to perform bacterial culture experiment to determine *Mycobacterium tuberculosis*. The presence of Mycobacteria (+) in a bacterial culture experiment is the gold standard for the diagnosis of lymph node tuberculosis. A pathological examination was required if the result of bacterial culture experiment was Mycobacteria (−). The pathological diagnosis is the gold standard for the diagnosis of non-tuberculosis lymph node.

The evaluation for probable supervifical TB lymphadenitis was as follows: (1) palpable superficial lymphadenectasis of the volume in patients was found in palpation or the ratio of length diameter to transverse diameter of the largest section of superficial lymph node was found below < 2, while measured in routine two-dimensional ultrasound examination [8]; (2) Color Doppler ultrasound examination; (3) Elastography examination; (4) Contrast-enhanced ultrasound examination; (5) puncture biopsy for bacterial culture experiment; (6) pathological examination if necessary.

Color Doppler examination: A color Doppler ultrasonic diagnostic apparatus (Philips iu22 Production address 22100 Bothell Everett Highway, Bothell, WA, 98021-8431 USA and Philips iu elite Production address 22100 Bothell Everett Highway, Bothell, WA, 98041-3003 USA) combined with broadband linear array probe (L12-5), with a frequency of 5.0–12.0 MHz was used. Ultrasound contrast agent was obtained from Sonovi (Brazil, Italy); its main component was sulfur hexafluoride. Distribution of the color Doppler flow in the lymph nodes was observed, and the results of the flow distribution were divided into four types: no blood flow, marginal blood flow, mixed blood flow, or central blood flow [9, 10].

Elastography: The real-time dual-amp mode was used with the broadband linear array probe touching the area to be examined in the patient, then the probe was slightly pressurized until each section was stable for at least 3 s, and an elastic image was acquired. There are a great many ultrasound elastographic techniques including strain imaging, acoustic radiation force impulse imaging, shear-wave elasticity imaging, supersonic shear imaging and so on [11, 12]. In the elastography imaging analysis, blue indicated hardness, and red indicated less hardness. The images were divided into types I–V [13, 14]: type I is red or yellow-green, type II is blue < 45%, type III is blue > 45%, type IV is blue in the peripheral zone and red or yellow-green in the center, and type V is blue throughout most of the lymph node.

Contrast-enhanced ultrasonography: (1) The largest section of the observed lymph nodes was selected. (2) The L9-3 broadband linear array probe with 3.0–9.0 MHz frequency was used in the ultrasound angiography; pulse-inversion harmonic imaging with a low mechanical index (MI) of 0.06 was used in the imaging. The contrast agent was obtained from Sono Vue (Bracco), diluted with 5 ml of physiological saline before use and shaken well, then 2.4 ml of the contrast agent was injected into the superficial elbow vein via bolus injection, followed by 5 ml of physiological saline. (3) Dynamic observation of the double-contrast imaging interface was used, and the timing and dynamic storage button was pressed when the contrast agent was input. Perfusion enhancement of the whole lymph node was observed for 3 min in real time, then the image of the entire imaging process was stored on the instrument hard disk. Superficial lymphography after superficial angiography is divided into five types: heterogeneity enhancement, uniformity enhancement, no enhancement, marginal and annular enhancement and separation enhancement [15, 16].

Assessment of multimodality: The multimodal evaluation methods in terms of color Doppler ultrasound, elastography, and contrast-enhanced ultrasound. The sensitivity, specificity and accuracy were calculated of color Doppler ultrasound and elastography. The sensitivity, specificity and accuracy were calculated of all three methods.

### Statistical analysis

Data analysis was performed using SPSS 23.0 statistical software. The measurement data are expressed as the mean ± standard deviation (x ± s) and were compared via t-tests. Countable data were expressed as the rate (%), and the two groups were compared using a *χ*^2^ test. Diagnostic tests were performed using sensitivity, specificity, positive, and negative predictive values, accuracy, and area under the receiver operating characteristic (ROC) curve. Differences were considered statistically significant at P < 0.05.

## Results

### General information

Baseline demographics of recruited subjects was shown in Table 1. A total of 60 patients with a mean age of 43.55 ± 19.21 years (ranged from 18 to 75 years) were finally selected in the study. Among the 60 cases, 38 (63.3%) were TB lymphadenitis and 22(36.7%) were non-TB lymphadenitis. Among the 38 patients with TB lymphadenitis, 10 were men and 28 were women, and the mean age was 52.64 ± 20.38 years (ranged from 18 to 68 years). Of the patients without TB lymphadenitis, 15 were men and 7 were women, and the mean age was 38.29 ± 16.59 years (ranged from 19 to 75 years). The 22 non- tuberculous cases included five cases of reactive proliferative lymph nodes (RPLN), four cases of normal lymphoid tissue (NLT), three cases of lymphadenitis (LA), two cases of Castleman's disease (CD), four cases of metastatic carcinoma (MC), one case of atypical cells (AC), and three cases of lymphoma (LO).

**Table 1:** Color Doppler ultrasound features in patients with and without superficial tuberculous lymphadenitis

Ultrasound color Doppler	Non tuberculosis [cases (%)]	Tuberculosis [cases (%)]	x^2^	P
Central type	11 (73.33%)	4 (26.67%)	10.048	0.002
Mixed type	6 (23.08%)	20 (76.92%)		
Marginal type	1 (11.11%)	8 (88.89%)		
No blood flow type	4 (40.00%)	6 (60.00%)		

### Characteristics of multimodal ultrasound imaging parameters in patients with and without superficial tuberculous lymphadenitis

**Fig. 1:**
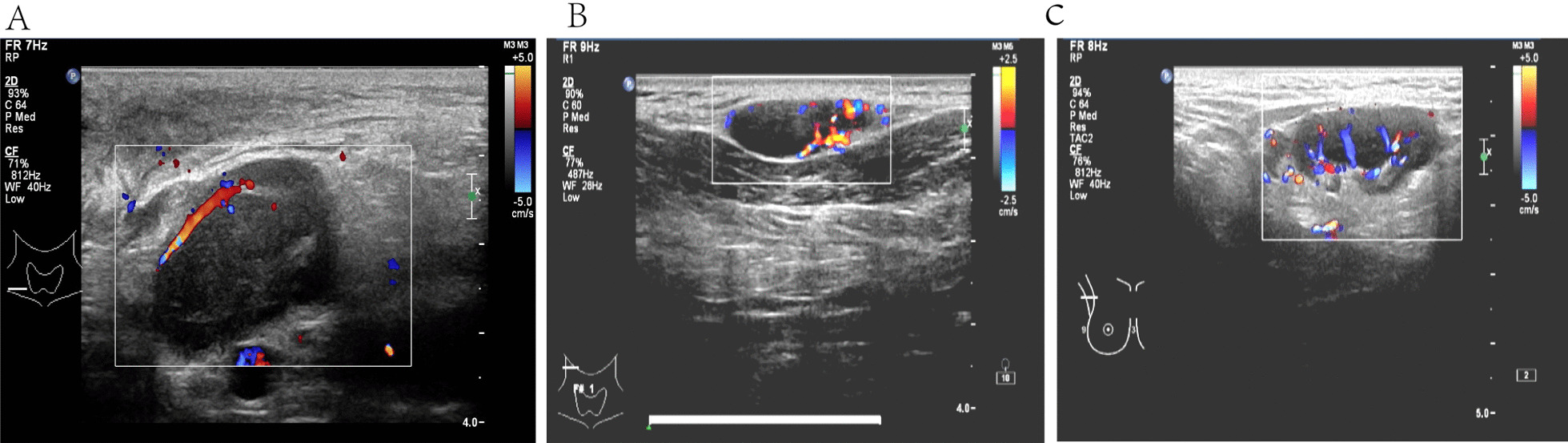
**A** Color Doppler flow image of tuberculosis in the right cervical lymph nodes, strip-shaped blood-flow signals could be seen at the margins. **B** Color Doppler flow image of tuberculosis in the right cervical lymph nodes, mixed-type blood-flow signals. **C** Color Doppler flow image of a patient with lymphadenitis in the right axillary lymph node, central-type blood-flow signals

**Fig. 2:**
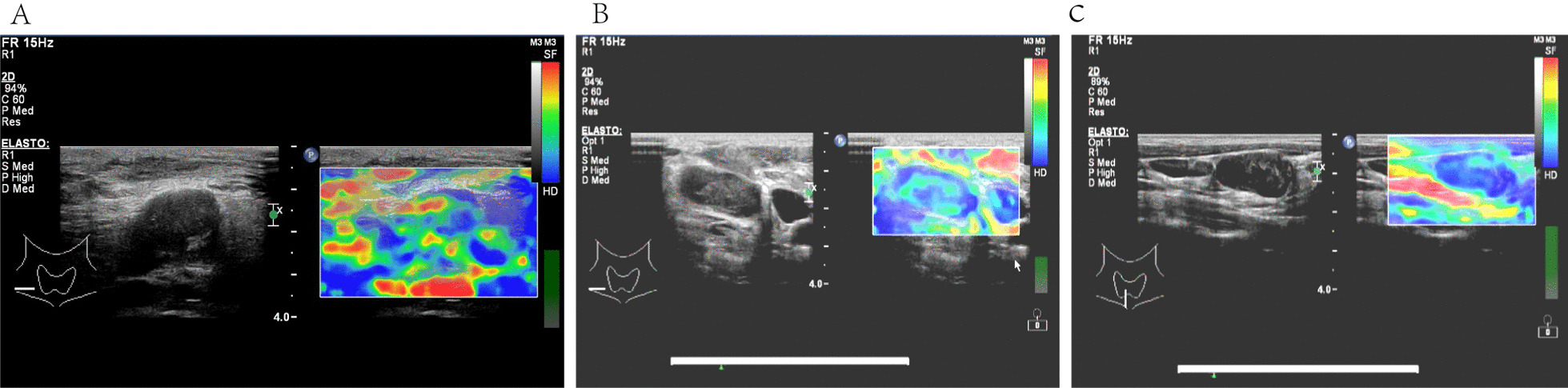
**A** Ultrasound elastography imaging of tuberculosis in the right cervical lymph nodes, type II. **B** Ultrasound elastography imaging of tuberculosis in the right cervical lymph nodes, type IV. **C** Ultrasound elastography imaging of tuberculosis in the right cervical lymph nodes, type V

**Fig. 3:**
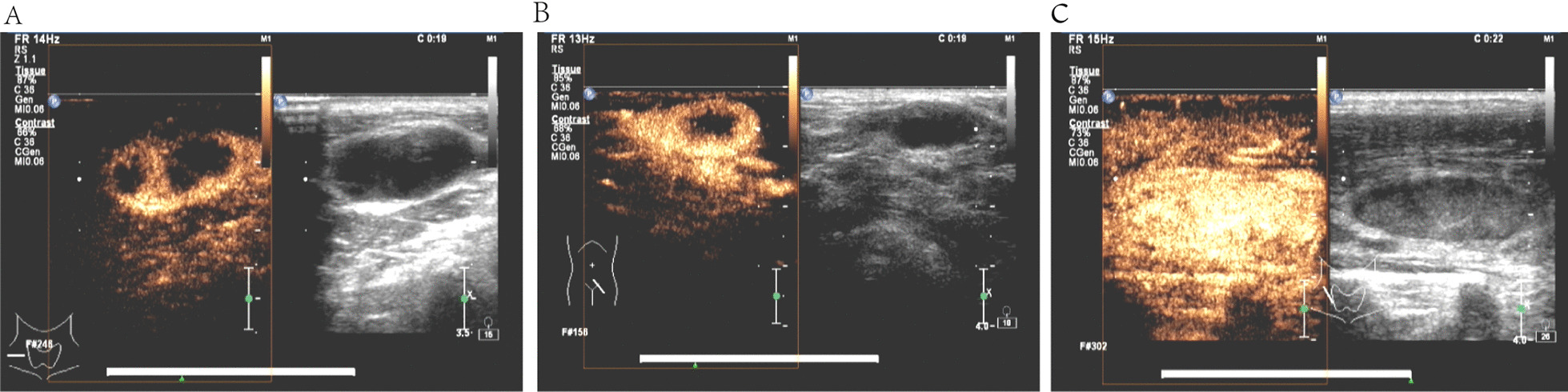
**A** Contrast-enhanced image of tuberculosis in the right cervical lymph nodes, separating enhancement. **B** Contrast-enhanced image of tuberculosis in the left inguinal lymph nodes, annular enhancement. **C** Contrast-enhanced image of lymphadenitis in the right cervical lymph nodes, uniformity enhancement

Baseline demographics of recruited subjects (Table 1). The blood flow distribution observes with superficial tuberculous lymphadenitis was mostly marginal (Table 2, Fig. 1). Most superficial tuberculous lymphadenitis cases were type II in the elastography imaging analysis (Table 3, Fig. 2). Lymphatic tuberculosis often 5 manifests as separation enhancement or annular enhancement (Table 4, Fig. 3). The key ultrasound features in tuberculous and non- tuberculous superficial lymphadenitis patients are summarized in Table 5.

**Table 2:** Elastography ultrasound imaging features of patients with and without superficial tuberculous lymphadenitis

Elastography ultrasound imaging features	Non tuberculosis [cases (%)]	Tuberculosis [cases (%)]	x^2^	P
Type I	1 (50.00%)	1 (50.00%)	4.019	0.045
Type II	7 (23.33%)	23 (76.67%)		
Type III	7 (53.85%)	6 (46.15%)		
Type IV	5 (45.45%)	6 (54.54%)		
Type V	2 (50.00%)	2 (50.00%)

**Table 3:** Ultrasonographic imaging features of patients with and without superficial tuberculous lymphadenitis

Ultrasound imaging features	Non tuberculosis [cases (%)]	Tuberculosis [cases (%)]	x^2^	P
Annular enhancement	3 (18.75%)	13 (81.25%)	18.715	< 0.001
Separating enhancement	0 (0.00%)	13 (100.00%)		
Uniformity enhancement	13 (76.47%)	4 (23.53%)		
Heterogeneity enhancement	5 (38.46%)	8 (61.54%)		
No enhancement	1 (100.00%)	0 (00.00%)

**Table 4:** Diagnostic value of color Doppler ultrasound, elastography and contrast-enhanced multimodal ultrasound for detecting superficial tuberculous lymphadenitis

Multimodal feature score	Sensitivity (%)	Specificity (%)	Positive predictive value (%)	Negative predictive value (%)	Accuracy (%)	Area under ROC curve
Color Doppler	73.68%	68.18%	80.00%	60.00%	71.67%	0.709
Elastography	63.16%	63.64%	75.00%	50.00%	63.33%	0.634
Contrast-enhanced ultrasound	89.47%	63.64%	80.59%	77.78%	80.00%	0.766
Color Doppler + elastography + contrast-enhanced ultrasound were all positive	42.11%	95.45%	94.12%	48.84%	61.67%	0.688
Either color Doppler + elastography + contrast-enhanced ultrasound is positive	100.00%	27.27%	70.37%	100.00%	73.33%	0.636

**Table 5:** Key ultrasound features in tuberculous and non-tuberculous superficial lymphadenitis patients

Ultrasound type	Tuberculosis	Non-tuberculosis
Color Doppler	1. Marginal blood flow 2. Heart-shaped blood flow	1. Hilus blood flow 2. No blood flow
Elastography	1. Red or yellow-green (type I) 2. Blue < 45% (type II)	1. Blue > 45% (type III) 2. Blue in the peripheral zone and red or yellow-green in the center (type IV) 3. Blue throughout most of the lymph node (type V)
Contrast-enhanced ultrasound	1. Marginal enhancement 2. Separation enhancement 3. Heterogeneity enhancement	1. Uniformity enhancement 2. No enhancement

### Diagnostic value of multimodal ultrasound imaging for superficial tuberculous lymphadenitis

The multimodal evaluation methods included color Doppler ultrasound, elastography, and contrast-enhanced ultrasound. All of these methods combined showed positive results for superficial tuberculous lymphadenitis, with 95.45% specificity and a 94.12% positive predictive value. Any of these alone showed positive results for superficial tuberculous lymphadenitis, with 100.00% sensitivity and a 100.00% negative predictive value (Table 6).

**Table 6:** Diagnostic value of color Doppler ultrasound, elastography, and contrast-enhanced multimodal ultrasound for detecting superficial tuberculous lymphadenitis

Multimodal feature score	Sensitivity (%)	Specificity (%)	Positive predictive value (%)	Negative predictive value (%)	Accuracy (%)	Area under ROC curve
Color Doppler	73.68%	68.18%	80.00%	60.00%	71.67%	0.709
Elastography	63.16%	63.64%	75.00%	50.00%	63.33%	0.634
Contrast-enhanced ultrasound	89.47%	63.64%	80.59%	77.78%	80.00%	0.766
Color Doppler + elastography + contrast-enhanced ultrasound all positive	42.11%	95.45%	94.12%	48.84%	61.67%	0.688
Either color Doppler + elastography + contrast-enhanced ultrasound is positive	100.00%	27.27%	70.37%	100.00%	73.33%	0.636

## Discussion

During the past two decades, the third global tuberculosis recurred because of the emergence and spread of drug-resistant mycobacterium tuberculosis [17]. In 2015, new cases of TB were reported to be 10.4 million globally, with six countries, India, Indonesia, China, Nigeria, Pakistan and South Africa, accounting for 60 percent of all the new cases [18]. International travel and residence for prolonged periods abroad have increased the risk of respiratory droplet spread of infection including drug-resistant TB, which can be mapped with molecular tools, and this has become an important way for the resurgence of tuberculosis. It was reported that, from January 1, 2014 to December 31, 2016, the detection rate of abnormal chest radiographs reached 14.70% among the 1721 immigrants from Fuzhou port to Canada [19]. These data suggest that TB remains to be a global concern.

Although the application of conventional ultrasound in the imaging of cervical lymph nodes has been well established [20], conventional two-dimensional ultrasound has no specific criteria for detecting tuberculosis of superficial lymph node. Multimodal ultrasound provides a more comprehensive assessment of superficial tuberculous lymphadenitis. Therefore, this study suggests the use of multimodal ultrasound evaluation methods to improve the value of ultrasound for diagnosing superficial tuberculous lymphadenitis.

Color Doppler ultrasound can be used to comprehensively explore the blood–flow signals and pathological changes in lymph nodes to provide a scientific and accurate reference for clinical medical treatment. In this study, 20 patients (76.92%) with superficial TB lymphadenitis exhibited a mixed blood flow. These results differed from those of Esen et al. [21]. This may be because different types of cervical lymph node tuberculosis manifest differently under Doppler ultrasound, while blood flow distribution in different types of tubercular lymphadenitis can occur in different lymph nodes within the same case. When the tubercular lymphadenitis exudates present peripheral inflammation, the corresponding pathological changes are mainly peri-lymphadenitis or tuberculosis infiltration, and adhesion to surrounding tissues [22], while the hilus remains intact. Thus, the center and periphery of a lymph node simultaneously show blood flow signals and present a mixed blood–flow distribution.

Elastography can be adopted to assess the hardness of superficial lymph nodes, thereby improving the ultrasonographic accuracy and it will be more helpful in diagnosing superficial tuberculous lymphadenitis [23, 24]. In this study, inter-reader variance of the elastography was avoided by performing assessment by at least three doctors. Twenty-three patients with superficial tuberculous lymphadenitis were type II, which was consistent with previous studies [25, 26]. When the lymph nodes were primarily blue on the elastography, the pathological specimens were mostly hard with granulomas and large amounts of caseous necrotic tissue. When the lymph nodes were primarily yellow-green on the elastography, the pathological specimens were soft with minimal caseous necrosis and heavily liquefactive necrosis.

Contrast-enhanced ultrasound can be used to evaluate the microcirculatory perfusion of superficial lymph node tissue. This study showed that superficial tuberculous lymphadenitis mainly appeared as annular enhancement and separation enhancement, which is related to the blood supply state at the margins and peripheries of the lymph nodes. There are three possible reasons for this: (1) *Mycobacterium tuberculosis* first accumulates in the lymph node tissue of the hilum, and the normal vascular structure is destroyed when cheese-like or liquefactive necrosis occurs, causing the center of the lymph node to lack blood–supplement action. (2) Much of the granulation tissue at the lymph node margin remains intact and becomes rich in new capillaries. (3) Granuloma formation in the marginal zone of the lymph node can induce an immune response in the surrounding soft tissues, and the inflammatory response –caused by the inflammatory cell infiltrate dilates the capillaries [27]. Here, we propose two methods for multimodal ultrasound evaluation of superficial tuberculous lymphadenitis. One method is the combination of color Doppler ultrasound, elastography, and contrast-enhanced ultrasound, which show positive results for superficial tuberculous lymphadenitis, with 95.45% specificity and 94.12% positive predictive value. The other is color Doppler ultrasound, elastography, or contrast-enhanced ultrasound individually, any of which shows positive results for superficial TB lymphadenitis. Each method alone has 100% sensitivity and 100% negative predictive value.

In this study, it was observed that contrast-enhanced ultrasound performed better than the combination of the three ultrasound examinations. In an ultrasound examination, anechoic or hypoechoic signal could be found inside a considerable proportion of superficial lymphatic tuberculosis, which indicates the liquefied necrosis or calcified areas in the lymph nodes. The former is mainly caused by *M. tuberculosis* entering the body and being engulfed by macrophages, and immune and delayed allergic reactions occur after 2 to 4 weeks, which results in tissue destruction. The latter is mainly caused by the cessation of the development of TB, which causes the deposition of calcium tissue, after which calcified areas are formed [28, 29]. Color Doppler ultrasound cannot show blood flow signals in the liquefied necrosis area or in the calcified area, but the same can be seen in contrast-enhanced ultrasound. The liquefied necrosis and calcified area in a superficial tuberculosis lymph node can also be observed via elastography. When there are a large number of areas with liquefied necrosis in the lymph node, the yellow-green signal appears in elastography; when there are many calcified areas in the lymph node, blue signal appears in elastography (inter-reader variance will occur when readers are variety in color perception, such as color weakness). However, sometimes these manifestations can only represent the areas of liquefaction or calcification but not the parenchymal portion of the lymph nodes. Therefore, contrast-enhanced ultrasound was required for higher accuracy in identifying tuberculous and non-tuberculous lymph nodes in this study.

Currently, color Doppler ultrasonography and elastography are very common ultrasound examinations, the processes of which are convenient and fast for superficial lymph nodes. Therefore, we recommend that the above two ultrasound examinations should be included in routine ultrasound examination for superficial lymph nodes. While contrast-enhanced ultrasound should be applied when color Doppler ultrasonography and elastography are still insufficient to diagnose the superficial lymph node because of its high accuracy. Moreover, contrast-enhanced ultrasound plays a directive role in guiding the ultrasound-guided biopsy. Thus, color Doppler, elastography, and contrast-enhanced ultrasound should be used as a complete ultrasound diagnosis system for superficial lymph node tuberculosis, as it would offer a high degree of specificity when the results of the three examinations are all positive.

A positive *M. tuberculosis* culture is the gold standard for the diagnosis of tuberculosis. Ultrasound examination is also useful in the differential diagnosis of tuberculous and non-tuberculous superficial tuberculous lymphadenitis for its high specificity; it also helps the clinician determine whether the patient really needs to undergo puncture biopsy. In addition, ultrasound monitoring is painless, easy to check, fast and inexpensive. Thus, ultrasound examination is recommended to be performed before a biopsy test, which may minimize the patient's pain. We believe this study will provide valuable information for the differential diagnosis of tuberculous and non-tuberculous superficial TB lymphadenitis.

Despite the interesting findings, our study still has certain limitations: (1) a small sample size, (2) a less detailed classification of superficial contrast findings of superficial tuberculous lymphadenitis, and (3) the lack of making multimodal ultrasound evaluation. Future research will attempt to remedy these defects in the present study.

## Conclusion

In summary, multimodal ultrasound imaging assessment was shown to have high specificity (with PPV and NPV range from 70.37% to 100%) in the differential diagnosis of tuberculous and non-tuberculous superficial tuberculous lymphadenitis, which may minimize the requirement of invasive diagnostic operation and contribute to the prognostic assessment of superficial TB lymphadenitis.

## Data Availability

Anonymized data can be made available to researchers who meet the conditions of the ethics approval and research governance policy that applies to this study. Researchers may request the data by contacting the corresponding author (yanggaoyi@163.com).

## Abbreviations

TB: Tuberculosis
ROC: Receiver operating characteristic
RPLN: Reactive proliferative lymph nodes
NLT: Normal lymphoid tissue
LA: Lymphadenitis
CD: Castleman’s disease
MC: Metastatic carcinoma
AC: Atypical cells
LO: Lymphoma
